# PS-11 - PILOTING AN ELECTRONIC VERSION OF A SHIGELLA ENHANCED SURVEILLANCE QUESTIONNAIRE ON ADULT CASES OF SHIGELLA FLEXNERI, LONDON 2024-25

**DOI:** 10.1093/pubmed/fdag046.060

**Published:** 2026-07-09

**Authors:** Miss Ellie Mackey, Ms Jill Bedesha, Ms Clare Sawyer, Ms Holly LeBlond, Mr Ed Andrews, Ms Charlotte Jackson, Dr Sooria Balasegaram

**Affiliations:** UK Health Security Agency; UK Health Security Agency; UK Health Security Agency; UK Health Security Agency; UK Health Security Agency; UK Health Security Agency; UK Health Security Agency

## Abstract

**Background:**

In England, local health protection teams (HPTs) assess laboratory confirmed Shigella flexneri cases. Cases complete enhanced surveillance questionnaires (ESQ) by phone to collect information for epidemiological trends and health protection action.

**Objectives:**

We began piloting an electronic questionnaire in London using an online platform in March 2024 with the aim to evaluate if online questionnaires impact response and any health inequalities.

**Methods:**

Case data from March 2024 – October 2025 was extracted from the online platform. We extracted a pre-pilot dataset of adult cases of Shigella flexneri cases from March 2023 – March 2024 from UKHSA case management data. We compared demographic characteristics (deprivation, age, sex, ethnicity and self-reported sexual orientation and contact). Deprivation was scored using small area measures of relative deprivation called indices of multiple deprivation (IMD).

**Results:**

The response rate was 69% (130/188) by phone and 39% (171/443) by electronic survey. The median age of responders for both groups was 37 years (18 – 84 for telephone respondents and 20 – 68 for electronic respondents). Sex distribution in each group was similar (83% male vs 79%). For telephone interviews, 70% reported being Gay Bisexual or other Men who have sex with Men, compared to 62% for electronic questionnaires. The median IMD scores were also similar (24.5 and 25.7). Information on ethnicity and recent sexual contact was more likely to be reported in electronic responses (99% and 95% vs 75% and 86%), X-squared 5.8, p = 0.02 for sexual contact.

**Conclusions:**

Proportionally more cases self-reported recent sexual contact when self-administering the questionnaire, suggesting cases may prefer this mechanism to disclose sensitive information. As the pilot is ongoing, further analysis will be undertaken to assess this finding and the characteristics of non-responders to improve online response. We recommend evaluating using electronic questionnaires as a baseline and following up non-responders by phone to improve completion.

